# Intrapartum rectovaginal distance as a predictor of obstetric anal sphincter injury: a prospective cohort study

**DOI:** 10.1590/1806-9282.20260531

**Published:** 2026-10-01

**Authors:** Elif Görkem Bademci, Yağmur Şimşek Erdim, Ecem Yüksel Özsoy, Erkan Elçi

**Affiliations:** 1 Sancaktepe Sehit Prof. Dr. Ilhan Varank Training and Research Hospital, Department of Gynecology and Obstetrics – Istanbul, Turkey.; 2 Sisli Hamidiye Etfal Training and Research Hospital, Department of Gynecology and Obstetrics – Istanbul, Turkey.; 3 Darıca Farabi Training and Research Hospital, Department of Gynecology and Obstetrics – Kocaeli, Turkey.; 4 Medicana Atakoy Hospital, Department of Gynecology and Obstetrics – Istanbul, Turkey.

**Keywords:** Obstetric labor complications, Perineum, Ultrasonography, Parity, Labor stage, Second

## Abstract

**OBJECTIVE:**

The aim of this study was to evaluate the relationship between intrapartum rectovaginal distance measured by transperineal ultrasonography and obstetric anal sphincter injury severity and second-stage labor duration in nulliparous women undergoing vaginal delivery.

**METHODS:**

This prospective cohort study was conducted at a tertiary referral center in Istanbul, between September 2021 and January 2022. Of 200 consecutive nulliparous women with singleton pregnancies at ≥37 weeks’ gestation, 148 who underwent vaginal delivery were included in the primary analysis. Intrapartum rectovaginal distance was measured in the midsagittal plane by 2D transperineal ultrasonography using a single operator during the inter-contraction rest period, at a fetal head station ranging from -1 to +1. Angle of progression and levator ani anteroposterior diameter were also recorded. Obstetric injury was graded per American College of Obstetricians and Gynecologists 2016 criteria; grade 3–4 laceration (severe obstetric anal sphincter injury) was the primary outcome.

**RESULTS:**

Severe obstetric anal sphincter injury occurred in 18 vaginal deliveries (12.2%). Rectovaginal distance was significantly shorter in women with severe obstetric anal sphincter injury (6.91±1.33 mm vs. 9.94±2.75 mm, p<0.001). Second-stage duration was longer with severe obstetric anal sphincter injury (125.8±6.5 vs. 91.3±18.0 min, p<0.001). Receiver operating characteristic analysis identified an rectovaginal distance cut-off of 8.1 mm (AUC=0.846; sensitivity 88.9%, specificity 80.8%). On multivariate logistic regression, rectovaginal distance (OR 0.516, 95%CI 0.373–0.713) and body mass index (OR 1.280, 95%CI 1.078–1.521) were independently associated with severe obstetric anal sphincter injury.

**CONCLUSION:**

Intrapartum rectovaginal distance measurement by transperineal ultrasonography is a noninvasive, readily obtainable parameter that independently predicts severe obstetric anal sphincter injury and may support individualized intrapartum risk stratification. Multicenter validation with larger samples is warranted.

## INTRODUCTION

Obstetric anal sphincter injury (OASI) represents the most clinically consequential complication of vaginal delivery, with an incidence of approximately 3% across all births and nearly 6% among nulliparous women^1^. Affected women face substantially elevated risks of fecal incontinence, dyspareunia, and chronic pelvic pain, with symptoms frequently remaining latent for decades^2-4^. A short perineal body, instrumental delivery, and nulliparity have been identified as significant anatomical and obstetric risk factors^5^. Despite growing awareness, prospective identification of at-risk women before delivery has yet to be incorporated into routine practice.

Transperineal ultrasonography (TPUS) offers non-invasive, real-time evaluation of fetal head descent and pelvic floor parameters^6^. The rectovaginal distance (RVD)—the shortest sonographic interval between the posterior vaginal wall and the rectal mucosa—has emerged as a surrogate for perineal tissue reserve. Prior reports linking reduced RVD to higher-grade trauma derive predominantly from postpartum examinations, where edema, hematoma, and surgical repair confound tissue geometry^7,8^. Intrapartum measurement during the active second stage may therefore yield superior predictive validity.

This study aimed to evaluate the relationship between intrapartum RVD, measured by 2D TPUS, and severe OASI in nulliparous women undergoing vaginal delivery, and to establish a sonographic cut-off for clinical risk stratification. Secondary objectives included assessment of the associations between angle of progression (AoP), levator ani contractile reserve, and second-stage labor duration.

## METHODS

This prospective cohort study enrolled consecutive nulliparous women (singleton, ≥37 weeks) at a tertiary referral hospital between September 2021 and January 2022. Ethical approval was obtained (Istanbul Ümraniye Training and Research Hospital Ethics Committee, Decision No: 15878), and written informed consent was provided by all participants.

Inclusion criteria: nulliparous women aged 18–45 years with confirmed gestational age. Exclusion criteria: chronic disease; BMI >35 kg/m^2^; multiple pregnancy; prior uterine surgery; substance use; fetal anomalies; assisted reproductive technology (ART) conception; gestational age <37 weeks; non-cephalic presentation. Eligible women were consecutively enrolled and data recorded prospectively.

### Ultrasound evaluation

All TPUS measurements were performed by a single experienced operator [E.E.] using an Esaote MyLab Seven ultrasound system (Esaote S.p.A., Genova, Italy) equipped with a convex array transducer (SB2C41, 1–8 MHz), with the patient in the lithotomy position. RVD was measured in the midsagittal plane as the shortest distance between the rectal mucosa and the posterior vaginal wall at the anorectal junction during the second stage; the mean of three measurements was used. All measurements were obtained during the inter-contraction rest period at a fetal head station ranging from -1 to +1 (mean: -0.4±0.7), ensuring reproducibility and reflecting resting tissue thickness. For the purposes of receiver operating characteristic (ROC) analysis, the optimal RVD threshold for prediction of severe OASI was determined post hoc using the Youden index. AoP was defined as the angle between the long axis of the symphysis pubis and a tangential line to the deepest bony part of the fetal head, measured in the midsagittal plane^6^. An AoP of ≥120° was considered indicative of imminent delivery, while values <120° were classified as unfavorable for progression, based on previously published thresholds. Levator ani muscle function was assessed by measuring the anteroposterior diameter of the levator hiatus at rest and during maximal voluntary contraction in the midsagittal plane. Contraction strength was expressed as the proportional reduction in hiatal diameter: (rest diameter - contracted diameter)/rest diameter×100 (%)^9^. A reduction of ≥20% was considered indicative of adequate contractile function. Labor duration was defined as the interval from full cervical dilation (10 cm) to delivery of the fetus, corresponding to the second stage of labor.

Active pushing with Valsalva maneuvers was used throughout the second stage under continuous fetal heart rate monitoring. Hands-on perineal support was applied and episiotomy performed at the clinician's discretion. Perineal trauma was assessed clinically immediately following delivery and confirmed by postpartum TPUS, both performed by the same operator who conducted the intrapartum measurements. Injuries were classified per American College of Obstetricians and Gynecologists (ACOG) 2016 criteria. Severe OASI was defined as third- or fourth-degree injury and constituted the primary outcome. Episiotomy sites were assessed separately and excluded from the laceration grading system.

### Sample size and statistical analysis

The sample comprised consecutively available patients; post hoc power analysis (G*Power 3.1) estimated 94% power (α=0.05, Cohen's d=0.8). Continuous variables are reported as mean±standard deviation (SD) or median [interquartile range (IQR)]; categorical variables as n (%). Group comparisons used the Student t-test or Mann-Whitney U test, and chi-square or Fisher's exact test as appropriate. Multivariate logistic regression with severe OASI as the outcome was performed; variables with p<0.05 in univariate analysis were considered for inclusion, with a maximum of two predictors per the events-per-variable rule (18 events). Discriminative ability was assessed by ROC analysis with a Youden index–derived threshold. Significance: p<0.05.

## RESULTS

Of 272 women assessed, 72 were excluded (36 chronic disease, 24 BMI >35 kg/m^2^, 12 multiple pregnancy) and 200 enrolled. Of these, 52 cesarean deliveries were excluded; the final cohort comprised 148 vaginal deliveries. Mean maternal age was 25.0±5.6 years, gestational age 39.0±1.4 weeks, and BMI 28.0±3.7 kg/m^2^. Episiotomy was performed in 133 women (89.9%), and operative vaginal delivery (Kiwi Omnicup) in 13 (8.8%). Mean intrapartum RVD was 9.6±2.8 mm, AoP 108.1±13.4°, and resting levator ani diameter 52.1±8.9 mm.

Perineal trauma occurred in 130 women (87.8%), including 18 (12.2%) with severe OASI (11 third-degree, 7 fourth-degree). Severe OASI (grade 3–4) occurred in 18 women (12.2%). BMI, RVD, AoP, resting levator ani diameter, second-stage duration, Apgar scores, and operative delivery rate differed significantly between women with and without severe OASI (all p<0.05; Table 1). Gestational age, BPD, birth weight, levator ani contraction strength, and episiotomy rate were comparable between groups.

**Table 1:** Comparison of clinical, ultrasonographic, and neonatal characteristics between women with severe obstetric anal sphincter injury (grade 3–4) and those without severe injury (grade 0–2) among 148 nulliparous women undergoing vaginal delivery.

Variable	Severe OASI (n=18)	No/mild OASI (n=130)	p-value
Maternal age (years)	26.1±5.6	24.8±5.6	0.313
Gestational age (weeks)	38.6±1.0	39.0±1.4	0.117
BMI (kg/m^2^)	29.9±2.5	27.8±3.7	0.005*
Rectovaginal distance (mm)	6.91±1.33	9.94±2.75	<0.001*
Angle of progression (°)	102.2±10.2	108.9±13.7	0.006*
Rest levator ani (mm)	55.7±5.2	51.6±9.2	0.011*
Levator ani contraction (mm)	42.1±2.7	43.7±10.7	0.522
BPD (mm)	91.6±3.2	91.4±3.4	0.962
Birth weight (g)	3379±413	3196±402	0.053
Second-stage duration (minutes)	125.8±6.5	91.3±18.0	<0.001*
1st min APGAR	5.6±1.0	7.0±0.8	<0.001*
5th min APGAR	7.1±0.8	8.3±0.8	<0.001*
Episiotomy, n (%)	18 (100%)	115 (88.5%)	0.217
Operative delivery (Kiwi), n (%)	12 (66.7%)	1 (0.8%)	<0.001*

Data are presented as mean±standard deviation or number (percentage). Between-group comparisons were performed using the Mann-Whitney U test for continuous variables and Fisher's exact test for categorical variables. Statistically significant results are indicated with an asterisk (*p<0.05). Second-stage duration was defined as the interval from full cervical dilation to delivery of the fetus. BMI: body mass index; RVD: rectovaginal distance; AoP: angle of progression; BPD: biparietal diameter; APGAR: Apgar score; OASI: obstetric anal sphincter injury.

RVD demonstrated high discriminative ability for predicting severe OASI (AUC=0.846); the optimal cut-off was 8.1 mm (sensitivity 88.9%, specificity 80.8%) (Figure 1). On multivariate logistic regression, RVD (OR 0.516, 95%CI 0.373–0.713, p<0.001) and BMI (OR 1.280, 95%CI 1.078–1.521, p=0.005) were identified as independent predictors of severe OASI (Table 2).

**Figure 1:**
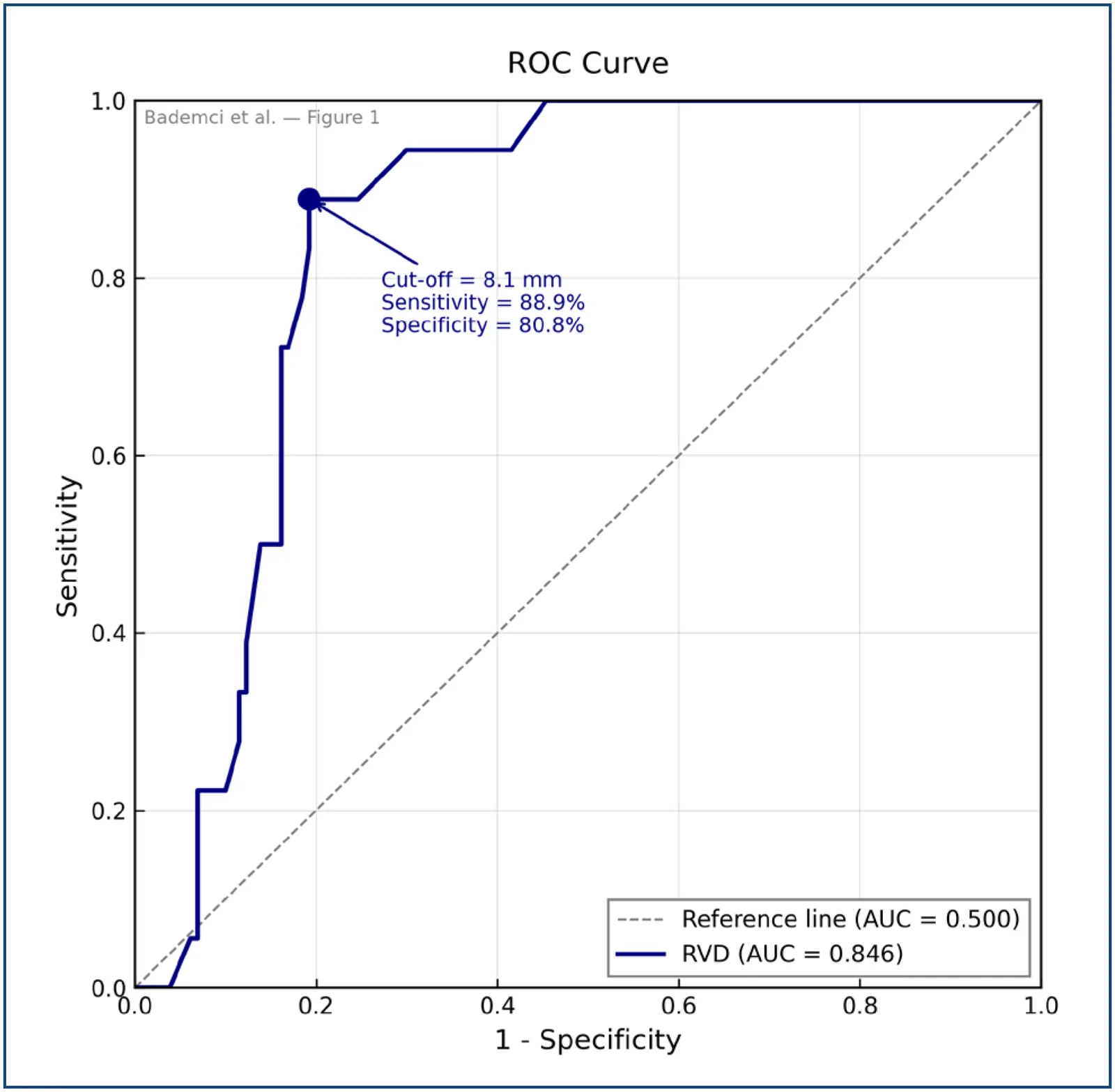
Receiver operating characteristic curve for intrapartum rectovaginal distance in predicting severe obstetric anal sphincter injury (grade 3–4). The optimal cut-off of 8.1 mm was determined by the Youden index (AUC=0.846; sensitivity 88.9%, specificity 80.8%). AUC: area under the curve; RVD: rectovaginal distance.

**Table 2:** Univariate and multivariate logistic regression analysis for predictors of severe obstetric anal sphincter injury (grade 3–4) among 148 nulliparous women undergoing vaginal delivery.

Variable		OR	95%CI	p-value
Panel A—Univariate analysis				
	RVD (mm)	0.594	0.461–0.765	<0.001*
	BMI (kg/m^2^)	1.165	1.020–1.330	0.025*
	Second-stage duration (minutes)	1.542	1.194–1.991	0.001*
	Operative delivery	258.0	28.6–2324.0	<0.001*
	AoP (°)	0.962	0.925–1.000	0.051
	Rest levator ani (mm)	1.055	0.996–1.117	0.069
	Birth weight (g)	1.001	1.000–1.003	0.075
	Levator ani contraction (mm)	0.984	0.937–1.034	0.528
Panel B—Multivariate logistic regression (Nagelkerke R^2^=0.362)				
	RVD (mm)	0.516	0.373–0.713	<0.001*
	BMI (kg/m^2^)	1.280	1.078–1.521	0.005*

Panel A presents crude odds ratios from univariate analysis; Panel B presents adjusted odds ratios from multivariate logistic regression including variables with p<0.05 on univariate analysis, limited to two predictors per the events-per-variable rule (18 events). Results are expressed as odds ratios (ORs) with 95%CIs. Statistically significant results are indicated with an asterisk (*p<0.05). Nagelkerke R^2^=0.362. BMI: body mass index; RVD: rectovaginal distance; AoP: angle of progression; OR: odds ratio; OASI: obstetric anal sphincter injury; CI: confidence interval.

## DISCUSSION

This study identified a clinically applicable intrapartum RVD threshold for severe OASI risk stratification. ROC analysis yielded an AUC of 0.846, with a cut-off of 8.1 mm achieving 88.9% sensitivity and 80.8% specificity. These metrics position intrapartum RVD as a useful screening parameter. The severe OASI rate of 12.2% observed in this cohort exceeds the 3–6% reported in general obstetric populations; this is attributable to the exclusively nulliparous study design, the high rate of operative vaginal delivery (66.7% in the severe OASI group), and the use of TPUS for injury detection, which has been shown to identify sphincter defects missed by clinical examination alone^10,11^.

Prior studies have linked reduced anovaginal tissue thickness to higher-grade perineal trauma; Pihl et al. and Hjertberg et al. demonstrated inverse relationships between RVD and OASI severity^7,8^. A 2023 meta-analysis by Packet et al. further identified perineal tissue dimensions and delivery mode as predictive factors^5^. The present study extends these observations by measuring RVD during active labor, avoiding the measurement variability introduced by postpartum edema and hematoma^10,12^, and establishes a precise numerical threshold for clinical use.

For women with an intrapartum RVD below 8.1 mm, evidence-based strategies including controlled fetal head delivery, hands-on perineal support, and selective mediolateral episiotomy may reduce sphincter injury risk^11,13^. Conversely, women with RVD exceeding 8.1 mm may not require routine episiotomy, consistent with current guidelines^11,14^. A 2025 systematic review confirmed that 2D TPUS in the delivery room has utility in assessing labor progress and recognizing OASI^15^.

A secondary aim of this study was to characterize the relationship between AoP and labor dynamics. In contrast to prior reports linking wider AoP to shorter labor duration^16,17^, AoP was not significantly associated with second-stage duration in our cohort (Spearman r=-0.119, p=0.149). This discrepancy may reflect the narrow range of AoP values observed during the active second stage, as measurements were obtained at a fetal head station ranging from -1 to +1, limiting variability in descent angle. However, AoP was significantly lower in women with severe OASI compared to those without (102.2° vs. 108.9°, p=0.006), suggesting that a more acute angle of fetal descent may be associated with greater perineal tissue loading, though this association did not reach independence in multivariate analysis. Descent mechanics and perineal tissue vulnerability thus appear to represent partially overlapping but distinct physiological domains.

Levator ani contractile function did not differ between women with and without severe OASI (p=0.522), consistent with Volløyhaug et al., who similarly found no early link between levator ani morphology and continence outcomes postpartum^18^. Pelvic floor dysfunction following OASI is recognized to follow a delayed trajectory, often manifesting decades after delivery as neurological and hormonal reserves decline^3^.

The limitations of the study include its single-center design, limited sample size, short follow-up period, and use of 2D measurements. Clinical anovaginal distance was not systematically recorded, precluding a direct comparison with sonographic RVD. The high institutional episiotomy rate (89.9%) reflects local practice patterns and limits generalizability to settings with selective episiotomy policies. Furthermore, the sample size, while adequately powered for large effect sizes, precludes construction of a robust multivariable prediction model. Notwithstanding these limitations, the prospective design, reduced group heterogeneity, and derivation of an RVD cut-off constitute the study's major strengths. Additionally, both intrapartum RVD measurement and postpartum OASI assessment were performed by the same operator, introducing the possibility of observer bias; blinded outcome assessment would strengthen future studies.

## CONCLUSION

Intrapartum RVD measured by TPUS is a non-invasive, readily obtainable parameter that independently predicts severe OASI and may support individualized risk stratification during the second stage of labor. Routine intrapartum RVD assessment may complement existing clinical tools for perineal injury prevention in nulliparous women. Multicenter validation with larger samples is warranted before widespread clinical implementation.

## Data Availability

The datasets generated and/or analyzed during the current study are available from the corresponding author upon reasonable request.
